# A Cross-Sectional Study of Quality of Life in Patients Enrolled in the Romanian Hereditary Angioedema Registry

**DOI:** 10.7759/cureus.51959

**Published:** 2024-01-09

**Authors:** Valentin Nadasan, Andreea Nadasan, Réka Borka-Balás, Noemi Bara

**Affiliations:** 1 Hygiene, George Emil Palade University of Medicine, Pharmacy, Science, and Technology of Targu Mures, Targu Mures, ROU; 2 General Medicine, George Emil Palade University of Medicine, Pharmacy, Science, and Technology of Targu Mures, Targu Mures, ROU; 3 Pediatrics, George Emil Palade University of Medicine, Pharmacy, Science, and Technology of Targu Mures, Targu Mures, ROU; 4 Allergy and Immunology, Hereditary Angioedema Expertise Center, Sangeorgiu de Mures, ROU

**Keywords:** pandemics, rare genetic diseases, quality of life, hereditary angioedema, c1-inhibitor

## Abstract

Background: Hereditary angioedema (HAE) is a rare potentially life-threatening genetic disorder characterized by recurrent episodes of angioedema without wheals that can affect any part of the body. The unpredictability of the attacks and the risk of passing the disease to the offspring result in significant physical and emotional burdens for patients, with a negative impact on quality of life. Data about the health-related quality of life in HAE patients from Romania are scarce. This study aimed to evaluate the disease-specific quality of life in patients with HAE from Romania and to determine associated factors.

Methods: The study included adult patients with HAE enrolled in the Romanian HAE Registry. Disease-specific quality of life was measured using the Hereditary Angioedema Quality of Life questionnaire, a cross-culturally adapted, internationally validated structured survey.

Results: The survey was completed by 94 patients (64.9% females; 35.1% males). The mean age of the participants was 44.9 years (SD 14.1). Most patients (88.3%) had type I HAE and were from urban areas (63.8%). The mean ages at symptom onset and diagnosis were 15.1 (SD 11.1) and 36.1 (SD 14.1) years, respectively. The mean diagnosis delay was 20.5 years (SD 14.2). In the evaluated period, all patients had at least one vial of on-demand treatment at home, and 10 were on long-term prophylaxis treatment. The general and dimensional quality of life scores were slightly above the median values of the reference scales. While the general score was not associated with sex or residence, a statistically significant, negative, weak correlation was detected with diagnostic delay.

Conclusion: The results suggest that despite the availability of on-demand treatment for all patients, there is a need for other diagnostic and therapeutic interventions to improve the management of the disease and the quality of life for HAE patients from Romania.

## Introduction

Hereditary angioedema (HAE) due to C1 esterase inhibitor (C1-INH) deficiency is a rare, life-threatening, autosomal dominant condition with an estimated prevalence of 1:50,000 to 1:67,000 in the general population [1]. Based on the global prevalence of this disease, it is assumed that there are an estimated 400 patients in Romania living with HAE. The condition is associated with a variety of mutations in the SERPING1 gene (some mutations have been identified in Romanian patients) responsible for deficient or dysfunctional C1-INH production [2,3]. A low C1-INH concentration leads to excessive levels of bradykinin, the main mediator of this type of angioedema [4]. The clinical picture is characterized by recurrent episodes featuring one or more of the following manifestations: swelling of the subcutaneous tissues of the limbs, face, or genitalia; swelling of the submucosal tissues in the gastrointestinal tract, frequently accompanied by severe pain; and swelling of the airways, sometimes leading to potentially life-threatening respiratory symptoms [5]. Untreated laryngeal edema in HAE patients is associated with a high risk of asphyxiation and, consequently, death [6,7]. Abdominal attacks may result in symptoms similar to those observed in acute abdomen, sometimes associated with ascites and hypovolemic shock [8]. Attacks related to C1-INH deficiency typically begin in childhood or adolescence, worsen during puberty, and persist throughout life. HAE symptoms mostly last two to five days before resolving spontaneously without any treatment. Between attacks, patients usually have no symptoms [9]. Because of its rarity, HAE often remains undiagnosed or misdiagnosed for years, which results in higher rates of morbidity and mortality [10].

While hereditary angioedema laryngeal attacks are potentially lethal, all attacks impose a considerable impact on patients’ quality of life. The unpredictability of the attacks and the risk of passing the disease to the offspring further amplify the psychological distress of the patients [6]. Moreover, a substantial proportion of HAE patients are misdiagnosed and undergo unnecessary, sometimes repeated, surgical procedures, especially during episodes with acute abdominal pain [11]. All these factors have a negative effect on the patients’ quality of life and contribute to the strain on the healthcare system in terms of direct and indirect healthcare costs [6].

HAE treatment involves both on-demand and prophylactic therapy, including short- and long-term prophylaxis [12]. Numerous treatment options are introduced worldwide, and some are available in Romania, including icatibant, plasma-derived C1-INH (pd-C1INH), and recombinant human C1-INH (rhC1-INH) for acute treatment and i.v. pdC1-INH for short- and long-term prophylaxis. Additionally, in cases of severe attacks, rhC1-INH or fresh frozen plasma (FFP) is available at county emergency departments (EDs) [13]. Although attenuated androgens and antifibrinolytic agents are unavailable in Romania, a few patients use them to prevent HAE attacks [14]. Kallikrein blocking agents such as ecallantide and berotralstat are not approved in Romania, and lanadelumab, a long-acting, fully human IgG1 monoclonal antibody with flexible subcutaneous dosing, became available in June 2022.

Assessing overall quality of life may provide insights into the management of the disease and provide a foundation for optimizing the management of patients with HAE. To date, information about the health-related quality of life in HAE patients from Romania is scarce and not based on validated, disease-specific questionnaires [14].

The study's primary objective was to assess disease-specific quality of life in adult patients from Romania with hereditary angioedema. The secondary objective was to explore the relationships between sociodemographic and disease-related characteristics on the one hand and quality of life on the other hand.

This article was previously presented as a meeting abstract at the 2022 International Conference of PhD Students and Young Doctors on December 7, 2022.

## Materials and methods

Study design, population, and sample

The research was designed as an observational (noninterventional) study. The study population comprised 120 hereditary angioedema patients recorded in the Romanian HAE registry. Eligibility was assessed based on inclusion and exclusion criteria.

Inclusion criteria: patients with a laboratory-confirmed diagnosis of hereditary angioedema with types I, II, and III; patients aged over 18 years old; patients with permanent residency in Romania; and patients providing informed consent. Exclusion criteria: patients aged less than 18 years old; patients with current symptoms of psychosis or dementia; patients with severe hearing impairment; and patients not able to provide informed consent.

**Figure 1:**
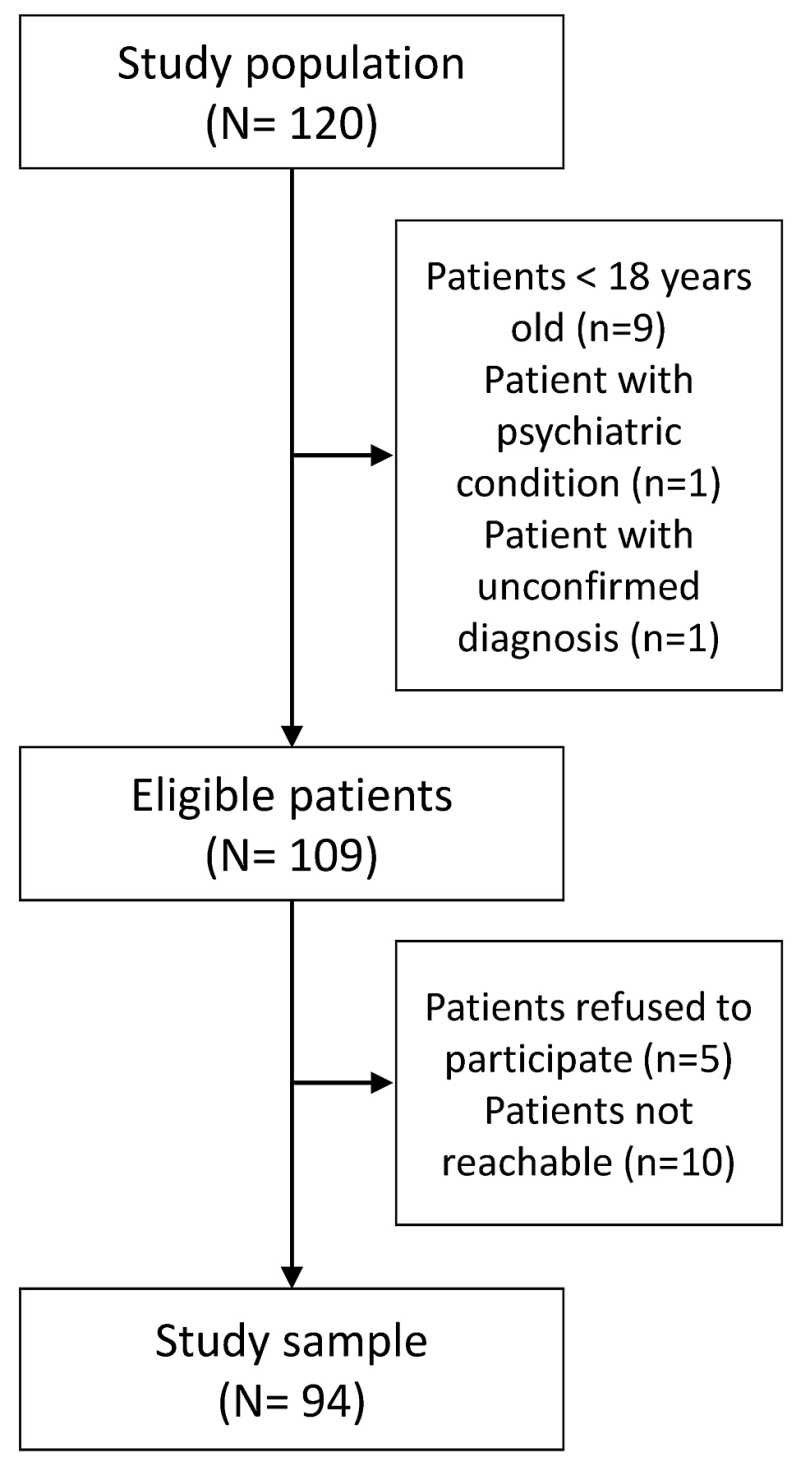
Study flow chart.

Numerical details about the study population, eligible patients, lost patients, and final study sample are represented in Figure 1.

Data sources, collection, and tools

Information regarding patient eligibility (Figure 1), basic sociodemographic characteristics such as age, sex, residency, type of hereditary angioedema, age at the onset of first symptoms, age at diagnosis, treatment availability, and frequency of attacks were collected from the Romanian Hereditary Angioedema Registry by authorized personnel in September 2021.

Data regarding disease-specific quality of life assessment were collected using a cross-culturally adapted and internationally validated structured survey, the Hereditary Angioedema Quality of Life Questionnaire (HAE-QoL) [15,16]. This tool has a six-month recall period and includes 25 Likert scale-type close-ended questions covering seven areas of quality of life that may be affected by the disease, such as physical functioning, physical role, social role, emotional role, symptoms, health conditions, esthetics, general health, mental health, and treatment. The patients’ quality of life is rated on a scale calculated based on the patients’ answers. Higher HAE-QoL scores correspond to a higher quality of life, and lower HAE-QoL scores correspond to a lower quality of life.

The Romanian version of the questionnaire was administered in September 2021 via telephone by trained personnel following detailed instructions. Patients received medications as prescribed by their treating physician, independent of this study.

Data analysis

Descriptive statistics were calculated (absolute and relative frequencies of nominal variables; mean and standard deviations of the numerical variables). The normality of numerical variables was checked using the Kolmogorov-Smirnov test of normality. The diagnosis delay was calculated by subtracting the age at the onset of symptoms from the age at diagnosis.

A composite, frequency-weighted disease severity score (DSS) was computed using the number of HAE attacks during the six months of the study and the patient-reported severity of the attack. Mild attacks, meaning attacks that had no impact on daily activities, were coded 1; moderate attacks, meaning attacks that partially limited the patient’s daily activities, were coded 2; and severe attacks, meaning attacks that completely prevented the patient from performing his/her daily activities, were coded 3. Each patient’s mean score was weighted based on the relative frequency of mild, moderate, and severe attacks, as shown in the following formula:

DSS = (1 × %Mi) + (2 × %Mo) + (3 × %Se),

where 1, 2, and 3 represent the codes attributed to mild, moderate, and severe HAE attacks, respectively; %Mi, %Mo, and %Se represent the relative frequency of mild, moderate, and severe HAE attacks reported by each patient during the six months of the study. Patients who did not report attacks during the study were included in the analysis with a DSS of 0 (zero).

The distance from the patient’s residence to the nearest hospital providing HAE-specific emergency care was computed using the Google Maps application based on the location of the patient’s residence and the nearest emergency hospital. The drive time in minutes was estimated at non-rush hours (10-12 a.m.). In the case of patients with no exact street address information, a city-wide mean distance and drive time were computed based on the distance from each urban sector to the nearest emergency hospital.

The HAE-QoL general and dimensional subscores were calculated according to the authors’ instructions. Questions with a five-level Likert scale were assessed using the following grading: Extremely=one point; Quite a lot=two points; Moderately=three points; A little=four points; Not at all=five points. For questions featuring a six-level Likert scale, the grading was as follows: All the time=one point; Most of the time=two points; Roughly half the time=three points; Some of the time=four points; A little of the time=five points; None of the time=six points. The general or global HAE-QoL score was calculated by summing the points assigned to all the questions. Dimensional scores were computed by summing the points from questions specific to each dimension as follows: Physical functioning and health: questions 1, 2, 3, and 19; Disease-related stigma: questions 4, 7, and 9; Emotional role and social functioning: questions 5, 6, 8, and 10; Concern about offspring: questions 11 and 12; Perceived control over illness: questions 13, 14, 18, and 25; Mental health: questions 15, 16, 17, and 23; and Treatment difficulties: questions 20, 21, 22, and 24.

Comparisons between subcategories by sociodemographic characteristics were performed using the student’s t-test or the Mann-Whitney test for numerical variables and the chi-square test or Fisher’s exact test for nominal variables. Correlations between numerical variables were analyzed using Pearson or Spearman correlation tests.

A multiple linear regression model was built to explain the variation in the HAE-QoL general score as the outcome variable. The candidate independent variables were determined to be those significantly correlated with the general score of HAE-QoL in this study, namely, diagnostic delay, distance to the nearest ED, and DSS. Additionally, age and sex were also considered independent variables, as they have been previously reported by other authors as significant predictors of quality of life in HAE patients [17,18]. Multicollinearity was checked to exclude predictors with correlation coefficients higher than 0.9. The fit of the regression model was assessed based on R square, R square change, and the significance of R square change.

Two-sided p-values were calculated, and the threshold for statistical significance was set at 0.05. Statistical analyses were performed using the Statistical Package for the Social Sciences Software (version 22.0, SPSS, Chicago, IL).

Ethical approval

The study was conducted in compliance with the requirements outlined in the Declaration of Helsinki and was approved in March 2021 by the IRB of the George Emil Palade University of Medicine, Pharmacy, Science, and Technology of Targu Mures, Romania (Decision No. 1306/19.03.2021). Informed consent was obtained by telephone at the first contact by the medical director as approved by the IRB of George Emil Palade University of Medicine, Pharmacy, Science, and Technology of Targu Mures, Romania.

## Results

The sample included 94 HAE patients. The sociodemographic and disease-related characteristics of the respondent and non-respondent patients are presented in Table 1, along with the results of the comparison tests to assess possible influences of a response bias on the study results.

**Table 1:** The socio-demographic, diagnostic, and therapeutical factors of respondent and non-respondent Romanian hereditary angioedema patients. ^a^: P-value of t-test for equality of means, independent samples; ^b^: P-value of chi-square test; ^c^: P-value of Mann-Whitney test; ^d^: P-value of Fisher's exact test; ED: emergency department, HAE: hereditary angioedema.

Variable	Respondents (N=94)	Non-respondents (N=15)	P-value
Age, years: mean (SD)	44.9 (14.1)	47.2 (14.4)	0.565^a^
Sex: n (%)			
Female	61 (64.9)	8 (53.3)	0.388^b^
Male	33 (35.1)	7 (46.7)	
Residence: n (%)			
Urban	60 (63.8)	10 (66.7)	0.831^b^
Rural	34 (36.2)	5 (33.3)	
Age at first symptom, years: mean (SD)	15.5 (11.1)	22.8 (17.8)	0.129^c^
Age at diagnosis, years: mean (SD)	36.1 (14.1)	40.4 (16.7)	0.287^c^
Diagnosis delay, years: mean (SD)	20.5 (14.2)	17.6 (13.4)	0.287^c^
HAE type: n (%)			
HAE type I	83 (88.3)	13 (93.3)	1.000^d^
HAE type II or III	11 (11.7)	1 (6.7)	
Received short-term prophylaxis: n (%)			1.000^d^
Yes	11 (11.7)	2 (13.3)	
No	83 (88.3)	13 (86.7)	
Received long-term prophylaxis: n (%)			1.000^d^
Yes	11 (11.7)	1 (6.7)	
No	83 (88.3)	14 (93.3)	
Received treatment in ED			1.000^d^
Yes	5 (5.3)	1 (16.7)	
No	89 (86.4)	14 (13.6)	

The mean number of attacks reported by a group of 30 patients (31.9%) was 13.8 (SD 12.6, minimum 0, maximum 44 attacks). The mean DSS in this subgroup was 1.8 (SD 1.0, minimum 0, maximum 3). No significant associations/correlations were observed between the number of attacks and sex, residence, HAE type, age, age at first symptoms, age at diagnosis, diagnosis delay, or distance to the nearest emergency department. Similarly, no associations/correlations were detected between DSS and sociodemographic and disease-related characteristics, except for age at diagnosis (Spearman rho=0.481, p=0.007) and diagnosis delay (Spearman rho=0.367, p=0.046). Compared to the full sample, the group of 30 patients with DSS data did not show statistically significant differences regarding sex (p=0.241), age (p=0.661), residence (p=0.695), or type of HAE (p=1.000).

During the study period, 93 (98.9%) of 94 patients had at least one vial of icatibant at their home, and five (5.3%) also used pdC1-INH for on-demand treatment. One patient (1.1%), a pregnant woman, used only pdC1-INH. In 11 (11.7%) patients, short-term prophylaxis was applied with pdC1-INH, and in one (1.1%) case, FFP was used for the same purpose. Long-term prophylaxis with pdC1-INH was applied in nine patients (9.6%), while attenuated androgens were administered in two patients (2.1%). In the reported period, five patients (5.3%) visited the ED for severe attacks and received additional treatment with rhC1-INH or FFP.

The mean distance from the patients’ permanent residence to the nearest emergency care hospital where HAE-specific treatment was available was 21.0 km (SD 18.6, minimum 1 km, maximum 73 km), while the mean drive time was 28.3 minutes (SD 18.6, minimum five minutes, maximum 75 minutes).

The HAE-QoL general score and dimensional subscores for the entire sample by sex, permanent residence, and HAE type are presented in Table 2. Higher scores correspond to a better quality of life. Reference values for the general score and dimensional subscores are reported in the last column.

Comparison tests of HAE-QoL general and dimensional scores in patients receiving vs not receiving short-term prophylaxis, long-term prophylaxis, and emergency medication in the ED did not reveal significant differences (p>0.05).

**Table 2:** General and dimensional HAE-QoL scores by sex, residence, and HAE type in 94 Romanian patients. HAE: hereditary angioedema; HAE-QoL: hereditary angioedema quality of life; ^a^: two-tailed P-value of t-test for equality of means, independent samples; ^b^: two-tailed P-value of Mann-Whitney test.

	Full sample (N=94); mean (SD)	Sex			Residence			HAE type			Reference values
		Female (N=61); mean (SD)	Male (N=33); mean (SD)	P-value	Urban (N=60); mean (SD)	Rural (N=34); mean (SD)	P-value	Type I (N=83); mean (SD)	Type II or III (N=11); mean (SD)	P-value	
HAE-QoL general score	78.0 (23.2)	76.4 (23.0)	81.1 (23.6)	0.352^a^	78.3 (23.8)	77.6 (22.5)	0.897^a^	79.1 (23.6)	70.3 (19.3)	0.239^a^	25-135
Physical functioning and health	12.6 (4.6)	12.6 (4.7)	12.7 (4.5)	0.946^b^	12.7 (4.8)	12.5 (4.5)	0.918^b^	12.9 (4.7)	11.0 (3.4)	0.167^b^	4-23
Disease-related stigma	9.8 (3.1)	9.5 (3.1)	10.3 (3.1)	0.175^b^	9.9 (3.1)	9.7 (3.2)	0.949^b^	9.9 (3.1)	9.1 (3.5)	0.401^b^	3-15
Emotional role and social functioning	13.0 (4.1)	12.9 (4.3)	13.3 (4.0)	0.656^a^	12.9 (4.0)	13.3 (4.4)	0.619^b^	13.2 (4.3)	11.7 (3.0)	0.144^b^	4-20
Concern about offspring	5.9 (2.5)	5.7 (2.4)	6.3 (2.8)	0.292^b^	5.9 (2.5)	5.9 (2.6)	0.975^b^	5.9 (2.6)	5.8 (2.4)	0.844^b^	2-10
Perceived control over illness	8.5 (3.7)	8.1 (3.6)	9.3 (3.9)	0.112^b^	8.6 (4.0)	8.4 (3.2)	0.899^b^	8.6 (3.8)	7.6 (3.0)	0.428^b^	4-20
Mental health	13.2 (4.9)	12.5 (4.7)	14.4 (5.2)	0.074^a^	13.1 (5.1)	13.3 (4.8)	0.843^a^	13.4 (5.1)	11.7 (3.5)	0.308^a^	4-24
Treatment difficulties	15.0 (4.9)	15.2 (4.9)	14.8 (4.9)	0.724^a^	15.3 (4.9)	14.6 (5.0)	0.495^b^	15.3 (4.9)	13.4 (4.9)	0.197^b^	4-23

The results of the correlation tests between the general and dimensional scores on the one hand and the age at the time of the study, age at first symptoms, age at diagnosis, diagnosis delay, and distance to the nearest emergency department on the other hand are reported in Table 3.

**Table 3:** Correlates of hereditary angioedema quality of life scores. HAE-QoL: hereditary angioedema quality of life; ED: emergency department; ^a^: Pearson correlation coefficient (r); ^b^: Spearman correlation coefficient (rho).

	Age		Age at first symptom		Age at diagnosis		Diagnosis delay		Distance to nearest ED	
	r/rho	P-value	r/rho	P-value	r/rho	P-value	r/rho	P-value	r/rho	P-value
General HAE-QoL score	−0.19^a^	0.072	0.18^b^	0.088	−0.19^b^	0.062	−0.32^b^	0.002	−0.24^b^	0.022
Physical functioning and health	−0.09^b^	0.365	0.10^b^	0.325	−0.15^b^	0.146	−0.20^b^	0.054	−0.31^b^	0.002
Disease-related stigma	−0.18^b^	0.091	0.20^b^	0.048	−0.12^b^	0.237	−0.31^b^	0.003	−0.20^b^	0.060
Emotional role and social functioning	−0.29^a^	0.005	0.26^b^	0.010	−0.22^b^	0.033	−0.40^b^	<0.0001	−0.11^b^	0.311
Concern about offspring	0.13^b^	0.227	0.34^b^	0.001	0.10^b^	0.347	−0.18^b^	0.076	−0.10^b^	0.350
Perceived control over illness	−0.18^b^	0.083	0.12^b^	0.263	−0.20^b^	0.056	−0.25^b^	0.014	−0.19^b^	0.075
Mental health	−0.18^a^	0.086	0.20^b^	0.055	−0.13^b^	0.200	−0.26^b^	0.012	−0.16^b^	0.114
Treatment difficulties	−0.19^b^	0.070	0.03^b^	0.754	−0.23^b^	0.028	−0.26^b^	0.011	−0.26^b^	0.013

The number of attacks and the disease severity score were significantly correlated with all quality-of-life scores, except for the “Concern about offspring” dimension. The detailed results of the correlation tests are presented in Table 4.

**Table 4:** The relationship between disease severity measures and hereditary angioedema quality of life scores. HAE-QoL: hereditary angioedema quality of life; ^a^: Pearson correlation coefficient (r); ^b^: Spearman correlation coefficient (rho).

Variable	Number of attacks		Disease severity score	
	r/rho	P-value	r/rho	P-value
General HAE-QoL score	−0.51^a^	0.004	−0.55^b^	0.001
Physical functioning and health	−0.42^a^	0.021	−0.57^b^	0.001
Disease-related stigma	−0.65^b^	<0.001	−0.52^b^	0.003
Emotional role and social functioning	−0.52^a^	0.003	−0.51^b^	0.004
Concern about offspring	−0.21^b^	0.263	−0.18^b^	0.331
Perceived control over illness	−0.45^b^	0.012	−0.46^b^	0.01
Mental health	−0.40^a^	0.029	−0.51^b^	0.004
Treatment difficulties	−0.50^b^	0.005	−0.49^b^	0.006

The results of the linear regression analysis are presented in Table 5.

**Table 5:** Multiple linear regression analysis in a group of 30 Romanian hereditary angioedema patients. DSS: disease severity score.

Predictors	Beta coefficient (95% CI)	Standardized beta	P-value	R^2^	Adjusted R^2^
DSS	−17.2 (−24.8 to −9.5)	−0.705	<0.001	0.445	0.403
Sex	18.7 (1.1 to 36.3)	0.332	0.039		

## Discussion

This is the first study to assess disease-specific quality of life in adult patients with HAE from Romania using a validated tool, HAE-QoL. The data covering six months of the second year of the COVID-19 pandemic may serve as a reference for international comparisons and a valuable baseline assessment for further HAE quality-of-life monitoring at the national level.

This research found general and dimensional HAE-QoL scores just slightly above the median values of the reference scales. While the general HAE-QoL score was not associated with sex, residence, short-term, long-term prophylaxis, or emergency medication administered in the ED, a statistically significant, negative, weak correlation was detected between the general HAE-QoL score on the one hand and diagnosis delay and the distance to the nearest ED on the other hand. Statistically significant but weak, positive, or negative correlates were observed for several HAE-QoL dimensional scores (Table 3). Older age was correlated with poorer emotional-social function. Higher age at first symptom was also correlated with poorer emotional-social function, and besides, with experiencing more disease-related stigma, more intense concerns about offspring, more frequent complaints regarding mental health. Higher age at diagnosis correlated with poorer emotional-social function and treatment difficulties. Longer diagnostic delays correlated with more disease-related stigma, poorer emotional-social function, poorer perception regarding the control of the illness, poorer mental health, and more treatment difficulties. Finally, greater distance to the nearest ED corresponded with more treatment difficulties. It is worth noting that while all these statistically significant correlations had a low strength, their direction (positive or negative) met the logical expectations of the questionnaire’s content.

The general score and all the dimensional scores except for “Concern about offspring” were inversely correlated, to a moderate or weak degree, with the number of HAE attacks and the DSS in an analysis of data from a group of 30 HAE patients.

The multiple linear regression model suggests that patients with increased disease severity scores have lower HAE-QoL scores (a severity score increase of one point predicted an HAE-QoL decrease of 17.2 points when controlling for the other variables). Additionally, the model predicted that males had an 18.7-point increase in the HAE-QoL score compared to females when other predictors were kept constant. Overall, the model with these predictors explained 44.5% of the variance in the total HAE-QoL general scores.

Romanian HAE patients seem to have sensibly lower general HAE-QoL scores (78.0) than patients in the USA (93.1) and Canada (102). Additionally, Romanian HAE patients show poorer dimensional scores than those in the USA and Canada [17,19]. These differences cannot be attributed to a lack of medication, as on-demand, short- and long-term HAE medication was practically available to all patients during the study period. The poorer HAE-QoL scores could be partially attributed to a combination of sociodemographic and clinical differences between Romanian patients and those from Canada and the USA. Our subjects had a higher mean age at diagnosis (36 years in Romania vs 20.0 years in Canada and 20.1 years in the USA) and a substantially longer diagnosis delay (20 years in Romania vs eight years in Canada and 8.4 years in the USA) [17,19]. Poor HAE-QoL scores in the Romanian sample may also be related to lower adoption (only nine patients) of inconvenient intravenously administered long-term prophylaxis (pd C1-INH) and poor control of the disease (in eight out of nine patients under long-term prophylaxis) attributed to insufficient dose or frequency of administration due to legal constraints (unpublished data from the Expertise Center). Other possible differences may be related to lower health literacy, deficiencies in patient-physician communication, more frequent misdiagnoses, and unnecessary treatments, as documented in a previous paper by Romanian authors [14].

Patients’ cultural norms and attitudes, such as acceptance of and resignation to disease and suffering, were suggested as explanatory factors of long diagnosis delay and could also contribute indirectly to lower quality of life [20]. Finally, we can speculate that the context of the COVID-19 pandemic may have had a significant negative impact on the quality of life of the patients studied. A recent study showed that patients with HAE perceived increased difficulties in accessing care, which, in turn, may influence their quality of life [21].

The negative correlation between quality of life and disease severity found in the present study aligns with the results reported in many countries using more or less similar assessment methodologies. An increased number of attacks was associated with lower HAE-QoL scores in HAE patients from Canada and the USA [17,19]. Regarding dimensional scores, our study found significantly lower dimensional scores in patients with more frequent HAE attacks except for the “concern about offspring” subscale, as reported by Banerji et al. [19]. In addition to HAE-QoL, various other measures of disease activity or severity were also correlated with a lower quality of life, heavier disease burden, higher level of anxiety or depression and stronger impact on daily activities and work in Europe [22,23], Japan [24], and Puerto Rico [18]. However, studies on HAE patients from China [25], Brazil [26], and Denmark [27] did not detect significant relationships. The lower quality of life in female HAE patients observed in our regression model (but not in the simple test of association) was reported by several authors using various quality-of-life assessment tools [17,18,25,28]. Older age was not a significant predictor of HAE-QoL scores in our regression model. Data regarding the relationship between age and disease-related quality of life in HAE patients are inconsistent. Similar to our results, some authors have found no significant correlation at all or a weak, positive relationship [17,25].

While a qualitative study suggests that patients in the USA having at-hand treatments that may be administered on-the-go do not worry so much about the proximity of medical facilities [29], our study showed that a longer distance to the nearest ED offering specific HAE emergency services was negatively, although weakly, correlated with the general HAE-QoL score. This observation corroborates the link between the longer time needed to reach the nearest hospital and the lower quality of life reported by other authors [17].

Study limitations and further studies

The study's potential limitations are related to sample size, selection of the patients, and data collection methods (via phone). A response bias regarding the HAE-QoL data is unlikely, as 94 out of 109 patients participated in the study (response rate 86.2%), and no significant differences regarding sociodemographic or disease-related characteristics were detected (Table 1). Additionally, the representativity of the overall study sample is probably unaffected even when considering the patients who were not invited to participate because of exclusion criteria (nine underage patients, one adult with psychiatric disease, and one adult lacking laboratory confirmation of the disease). The regular submission of the patient diaries containing data regarding the number of attacks was disrupted during the pandemic, and as a result, the analyses of disease severity in relation to HAE-QoL were limited to a subsample of 30 patients. While the reduced size of the subsample may question the generalizability of the results regarding disease severity and its relationship to HAE-QoL scores, comparison tests did not reveal significant sociodemographic differences between this group and the rest of the sample.

Recall bias, a limitation inherent to collecting data about symptoms and events experienced in the past, was minimized by using the HAE-QoL, a tool that was validated for the Romanian language, and by adapting the pace of the survey to the individual reaction time of the respondents. Finally, social desirability bias, generally associated with face-to-face and telephone interviews conducted by individuals known to the respondent, was addressed by assuring the participants that their answers would be anonymized, disclosed only in an aggregate form, and by employing interviewers unknown to the patients.

As the Romanian HAE Registry continues to grow and enroll more new patients, future studies will hopefully ensure increased statistical power. Further studies should explore additional relevant predictors such as comorbidities and different types of medications used for long-term prophylaxis, such as lanadelumab, recently approved in Romania as self-administered subcutaneous injection home treatment.

## Conclusions

The quality of life of HAE patients from Romania during the second year of the COVID-19 pandemic was poor, as measured using the HAE-QoL questionnaire. The general HAE-QoL score and all, except one of the dimensional scores, were negatively correlated with the number of HAE attacks and disease severity score. The multiple linear regression model, including disease severity and sex, could predict more than 44.5% of the variance in the general HAE-QoL scores. The results suggest a large room for improvement in managing HAE in Romania.
